# Polysubstance Use After Frontal Lobe Syndrome: A Case Report

**DOI:** 10.1192/j.eurpsy.2022.2142

**Published:** 2022-09-01

**Authors:** M. Ulusoy, M. Ergelen

**Affiliations:** 1 Ankara University Faculty of Medicine, Department Of Psychiatry, Ankara, Turkey; 2 Istanbul Erenkoy Training and Resarch Hospital for Psychiatry and Neurological Diseases, Department Of Psychiatry, Istanbul, Turkey

**Keywords:** substance abuse, frontal lobe syndrome, traumatic brain injury

## Abstract

**Introduction:**

Frontal lobe syndrome (FLS) is a clinical condition characterized by personality and behavioral changes that usually occur after a traumatic brain injury (TBI). The main features of this syndrome are related to the deterioration of basic functions of the frontal lobe. Substance use disorder (SUD) is rare but also serious comorbiditiy seen after TBI.

**Objectives:**

In this case report, we aimed to discuss a case who developed SUD after TBI.

**Methods:**

Case report

**Results:**

A 40-year-old male patient with history of using cannabis, methamphetamine, synthetic cannabinoid was admitted to our alcohol and substance use disorders research and treatment centre (AMATEM) inpatient unit for detoxification. He has reported that he was injured by a car accident five years ago, had a surgery and was hospitalized for a few months, and started to use substance to relieve pain. According to the medical records, the left frontal and temporoparietal regions were affected. He reported no history of substance abuse before injury, no previous history of psychiatric admission. Personality and behavior changes had been observed after TBI. In the first examination he had depressed mood and loss of interest. Sertraline (gradually titrated up to 150 mg/d) and risperidone (1 mg/d) were started. Also *N*-acetylcysteine (1,200 mg/d) was added to reduce craving and drug-seeking behaviours for four weeks.

**Conclusions:**

Frontal lobe syndrome and TBI may differ in terms of clinical presentations. Substance use may be a way to cope with mental, cognitive or behavioural changes, psychosocial stressors, anxiety, sleep problems or pain after TBI.

**Disclosure:**

No significant relationships.

